# Extracellular vesicles in cardiac surgery: unlocking new frontiers in cardioprotection and patient outcomes

**DOI:** 10.1007/s10238-025-01945-z

**Published:** 2025-11-25

**Authors:** Alessandro Carrozzo, Ilenia Pia Cappucci, Laura Basile, Elena Tremoli, Barbara Zavan, Letizia Ferroni

**Affiliations:** 1 Maria Cecilia Hospital, GVM Care and Research, 48033 Cotignola, Italy; 2 Translational Medicine Department, University of Ferrara, 44121 Ferrara, Italy

**Keywords:** Coronary artery bypass grafting, Valve repair and replacement, Extracellular vesicles, Cardioprotection, Ischemia–reperfusion injury, Myocardial infarction

## Abstract

Cardiac surgery, while life-saving, induces profound physiological stress due to ischemia–reperfusion injury (IRI), systemic inflammation, and endothelial dysfunction, particularly in procedures involving cardiopulmonary bypass. In this complex setting, extracellular vesicles (EVs) have emerged as both biomarkers and potential mediators of cardiovascular injury and repair.

This narrative review explores the multifaceted roles of EVs in cardiac surgery, with a focus on coronary artery bypass grafting (CABG) and valve repair or replacement. The review examines the diagnostic and therapeutic implications of circulating EVs and their role in graft patency, perioperative complications, myocardial protection, and vascular remodeling.

We summarize current evidence regarding the biogenesis, classification, and engineering of EVs, highlighting their ability to transport bioactive molecules that modulate inflammation, coagulation, and apoptosis. In CABG, EVs have been linked to systemic inflammatory response, myocardial injury, and postoperative cognitive dysfunction. In valvular heart surgery and transcatheter procedures, endothelial- and platelet-derived EVs correlate with endothelial injury, shear stress, and postoperative outcomes. Preclinical studies indicate that stem cell-derived EVs exert cardioprotective effects by reducing apoptosis, promoting angiogenesis, and reprogramming macrophages.

EVs represent a promising frontier in cardiac surgery, offering opportunities for risk stratification, real-time monitoring, and novel therapeutic strategies. Further translational research and standardized clinical protocols are needed to integrate EV profiling into perioperative care and to explore the full potential of EV-based therapies in cardioprotection and vascular healing.

## Introduction

Cardiac surgery remains a cornerstone in the management of advanced cardiovascular diseases, particularly in patients with complex coronary artery disease (CAD) and valvular heart disease (VHD). Despite major technological and procedural advances, postoperative complications such as systemic inflammation, ischemia–reperfusion injury (IRI), thrombosis, and organ dysfunction continue to limit outcomes and increase healthcare burden. These adverse events are often driven by a complex interplay of molecular and cellular mechanisms that are not fully understood, highlighting the need for novel biomarkers and therapeutic targets to improve surgical outcomes and long-term prognosis [1, 2].

In this context, extracellular vesicles (EVs) have emerged as key players in cardiovascular pathophysiology. EVs are membrane-enclosed nanoparticles actively released by nearly all cell types in response to several signal such as stress, activation, or injury. Far from being inert byproducts, EVs mediate intercellular communication by delivering proteins, lipids, and nucleic acids to recipient cells locally or systemically [3–5]. In the perioperative setting of cardiac surgery, the release of EVs is dramatically enhanced due to the exposure of blood to artificial surfaces, ischemia–reperfusion cycles, and systemic inflammation induced by cardiopulmonary bypass (CPB). Several studies have shown that EVs derived from activated platelets, leukocytes, endothelial cells, and cardiomyocytes accumulate in the circulation during surgery and correlate with critical events such as endothelial dysfunction, thrombin generation, and inflammatory cytokine release [6–9]. For instance, platelet- and endothelial-derived EVs expressing tissue factor and phosphatidylserine amplify the coagulation cascade and contribute to a pro-thrombotic state, increasing the risk of graft occlusion and postoperative thromboembolic complications [10, 11]. Moreover, EVs are not merely markers of ongoing damage, as they can actively propagate it. They are involved in the pathogenesis of IRI, where they mediate oxidative stress, activate immune cells, and promote apoptosis in cardiomyocytes. EVs enriched in mitochondrial DNA or pro-inflammatory microRNAs (miRNAs) can amplify sterile inflammation and contribute to the development of systemic inflammatory response syndrome (SIRS) and postoperative organ dysfunction, including acute kidney injury and neurological impairment [12, 13]. Paradoxically, the same vesicles also hold great promise as diagnostic and therapeutic tools. EVs released during cardiac stress can carry cardioprotective signals, such as anti-apoptotic proteins or regenerative miRNAs, capable of limiting tissue damage and supporting myocardial recovery [14]. In fact, EVs secreted by progenitor cells or preconditioned cardiac tissues have demonstrated efficacy in experimental models of myocardial infarction and IRI, reducing infarct size and improving cardiac function [15, 16].

**Fig. 1:**
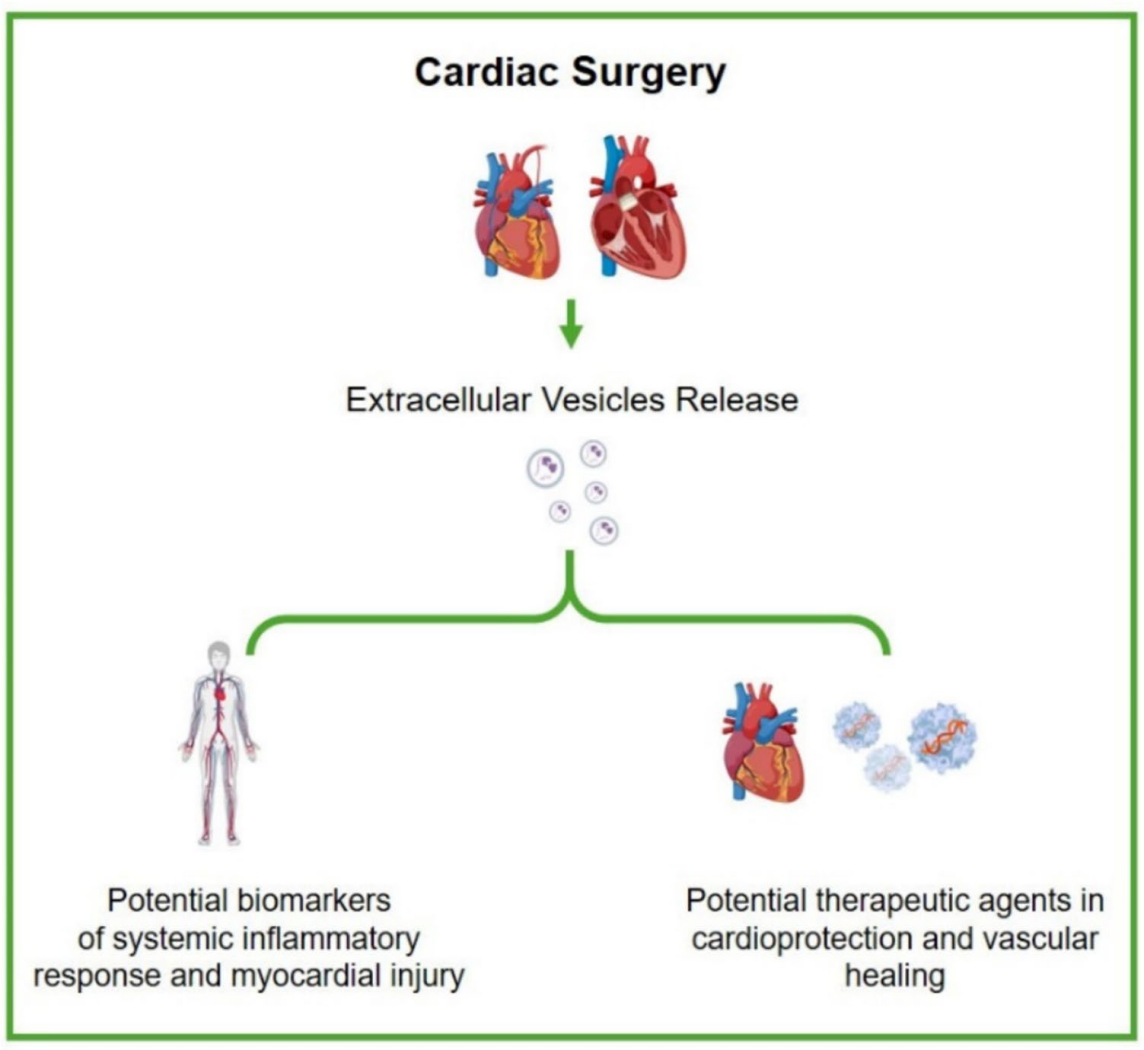
Dual role of extracellular vesicles (EVs) in cardiac surgery. EVs act both as mediators of myocardial injury and as carriers of therapeutic signals, highlighting their central role in the perioperative course

The dual function, as damage mediators and therapeutic vectors (Fig. 1), underlines the fundamental role of EVs in the perioperative course of cardiac surgery.

By profiling their concentration, origin, and molecular content, clinicians may gain valuable insights into the inflammatory status, coagulation dynamics, endothelial function, and even neurocognitive vulnerability of each patient. EVs hold significant promises as biomarkers for predicting and monitoring complications during and after cardiac surgery. For instance, changes in the concentration or molecular composition of circulating EVs reflect underlying endothelial health, inflammatory activity, or coagulation balance [17]. A deeper understanding of the multifaceted roles of EVs in cardiovascular pathophysiology might enable researchers and clinicians to design innovative strategies for improving patient outcomes. These may include tailored pharmacological approaches aimed at reducing the harmful effects of EVs while harnessing their potential for repair and recovery, ultimately advancing the field of cardioprotection. Furthermore, engineering EVs to deliver targeted therapies could open new avenues in precision cardioprotection, allowing the modulation of immune responses, enhancement of repair mechanisms, and personalization of perioperative strategies.

Therefore, this review aims to provide a comprehensive and critical overview of the current knowledge on EVs in the context of cardiovascular surgery. Special attention is given to their role in coronary artery bypass grafting (CABG) and valve procedures, their potential as clinical biomarkers, their involvement in adverse outcomes such as IRI, SIRS, and graft failure, and the future perspectives of EV-based therapeutic applications.

## Extracellular vesicles

EVs are defined as nanoscale bilayer phospholipid membrane vesicles released by cells in response to various physiological and pathological stimuli. EVs are active participants in intercellular communication, serving as vehicles for transporting bioactive molecules such as proteins, lipids, DNA, several type of RNA, and metabolites. Their role has been recognized as critical in both physiological regulation and progression of pathological conditions, particularly in the cardiovascular system [3, 4]. In accordance with the updated MISEV2023 guidelines [18], EVs should preferably be described using operational terms that reflect measurable physical or biochemical properties rather than presumed mechanisms of biogenesis. Therefore, EVs can be classified according to size as small EVs (diameter less than 200 nm), medium EVs (between 200 and 800 nm), and large EVs (higher than 800 nm) or by biochemical composition, density, or cellular origin. Nevertheless, for clarity and to maintain consistency with the majority of existing literature, in this manuscript we will continue to refer to exosomes (EXOs) and microvesicles (MVs), acknowledging that these terms primarily reflect their putative biogenesis rather than definitive or exclusive categories [18]. MVs typically are formed by direct outward budding from the plasma membrane in response to activation, stress, or apoptosis stimuli. They maintain the surface markers of their parent cells. For instance, in the bloodstream it is possible to distinguish endothelial, leukocyte, and platelet MVs, which play significant roles in coagulation, inflammation, and cellular communication [4]. EXOs are vesicles formed by inward invagination of the endosomal membrane and fusion of the multivesicular body with the plasma membrane. They display specific markers, including CD81, CD9, and CD63, and containing proteins, DNA, RNA, and miRNAs. They are involved in various cellular processes, including immune responses, cell signaling, and waste management [19]. Instead, apoptotic bodies are the largest vesicles (diameter from 1 to 5 μm) and are formed during apoptosis by outward blebbing of the cell plasma membrane [5].

Given their multiple functions, EV isolation and characterization are critical for understand their full potential in both research and therapeutic applications. EV separation relies on approaches which employ their physical and biological features such as size, density, charge, or surface markers [20]. Among these, differential ultracentrifugation is commonly used, pelleting MVs at 10,000–20,000 × *g* and EXOs at 100,000–200,000 × *g*, but it suffers from incomplete separation and contamination. Density gradient centrifugation separates EVs based on their density, effectively removing protein aggregates and lipoproteins. Alternatively, size exclusion chromatography uses porous beads to separate EVs from smaller soluble proteins and contaminants without damaging vesicle integrity, making it suitable for functional studies. Ultrafiltration concentrates EVs from a suspension using membrane filters. Finally, immunoaffinity-based isolation leverages antibodies targeting tetraspanins (*e.g.*, CD9, CD63, CD81) conjugated to magnetic beads to selectively enrich specific EV subtypes, though can miss certain EV subtypes or bind off-target. Each method balances trade-offs in purity, yield, resolution, throughput, and scalability [21]. Once isolated, characterization of EVs includes techniques such as nanoparticle tracking analysis or dynamic light scattering to determine their size distribution and concentration. Transmission electron microscopy provides detailed morphological information, confirming spherical structure typical of EVs. Additionally, flow cytometry and Western blotting are employed to identify specific protein markers on the EV surface, ensuring that the isolated vesicles represent the desired EV subpopulation. These methods, when combined, allow for a comprehensive analysis of EVs, essential for their application in biomarker discovery, drug delivery, and therapeutic strategies [18, 22].

To further enhance their therapeutic potential, EVs can be engineered through various strategies designed to optimize their cargo content, targeting ability, and functional performance. Broadly, EV engineering can be divided into endogenous and exogenous approaches [23]. In endogenous engineering, also known as passive pre-loading, the parental cells are genetically or pharmacologically manipulated to produce EVs with specific characteristics. This approach involves transfecting EV-secreting cells with synthetic molecules or miRNA-expressing plasmids and viral vectors, increasing intracellular levels of desired target which are then passively incorporated into EVs during biogenesis. This endogenous loading method is attractive because it preserves vesicle integrity and avoids direct structural disruption [24]. In contrast, exogenous engineering involves modifying EVs after their isolation. This includes techniques such as electroporation, sonication, freeze–thaw cycles, or chemical transfection to load EVs with therapeutic agents such as anti-inflammatory miRNAs, small interfering RNAs (siRNAs), or drugs. Additionally, surface functionalization strategies such as ligand conjugation or membrane fusion with targeting peptides can be employed to direct EVs specifically to injured cardiac tissue or inflamed vasculature, enhancing site-specific delivery and minimizing off-target effects. These engineering strategies are particularly valuable in the cardiovascular field, where precision and controlled delivery are crucial [25]. To illustrate the diversity and utility of these engineering strategies, Table 1 summarizes various methods for loading therapeutic agents into EVs, highlighting their respective advantages and limitations in the context of cardiovascular disease treatment.

**Table 1:** Different approaches to loading therapeutic agents into EVs

Method	Description	Advantages	Limitation	Refs
Chemical permeabilization	Use detergents like saponin to create transient pores in the EV membrane	Loading a variety of cargo types; relatively simple	Risk of damaging ev membrane if not carefully controlled	[26]
Electroporation	Apply an electric field to create temporary pores in EV membranes allowing small molecules to enter	High efficiency, scalable, preserves EV integrity	Potential membrane damage; aggregation of cargo	[27]
Extrusion	Forcing EVs through small pores to introduce cargo via mechanical pressure	High control over cargo-to-EV ratio	Risk of Ev fragmentation if not optimized	[28]
Incubation (passive loading)	Incubate purified EVs with therapeutic agents, allowing diffusion of cargo into EVs	Simple and straightforward; minimal equipment required	Low loading efficiency; may not work well for large molecules	[29]
Pre-loading via cell engineering	Genetically modify donor cells to express therapeutic proteins or RNA that are packaged into EVs	EVs remain intact, targeted release of cargo	Time-consuming; it requires genetic modification of cells	[30]
Sonication	Use ultrasonic waves to disrupt EV membranes temporarily, allowing cargo to enter	High loading efficiency; fast process	It can lead to EV damage and loss-of-functional integrity	[31]

## Extracellular vesicles and cardiovascular surgery: key challenges

### Coronary artery bypass graft surgery

CABG remains a fundamental intervention in patients with advanced CAD. Despite its proven benefits in improving survival and relieving symptoms, CABG is associated with significant systemic inflammation and IRI, particularly when performed with CPB. One of the most relevant postoperative complications is SIRS, which may lead to multiorgan dysfunction and prolonged recovery. SIRS in CABG unfolds in two phases: an early phase initiated by surgical trauma and endothelial disruption, and a late phase predominantly driven by IRI [32, 33]. Emerging evidence implicates EVs, particularly EXOs, in the modulation of these inflammatory responses (Table 2).

**Table 2:** Extracellular vesicles in cardiovascular surgery

Surgery	EV population	Outcome	Refs
CABG: 15 patients	EVs carrying cardiac miRNAs: miR-1, miR-24, miR-133a/b, miR-208a/b, miR-210	Plasma EV concentrations and cardiac miRNAs increased post-surgery correlating with cardiac Troponin I levels, indicating myocardial injury and EV release from the heart	[34]
CABG: 60 patients	p-EVs: CD41^+^, CD62P^+^/CD41^+^, CD40L^+^/CD41^+^ m-EVs: CD14^+^ g-EVs: CD66^+^ e-EVs: CD31^+^/CD41^−^	CABG-treated patients who will experience mid-term graft occlusion are characterized by a pre-surgery EV signature indicative of platelet activation status and supporting a greater thrombin generation capacity compared to that of patients with patent grafts	[11]
On-pump CABG: 18 patients Off-pump CABG: 18 patients	p-EVs:CD62P^+^ e-EVs: CD31^+^ Bc-EVs: CD19^+^	EV levels increased significantly after on-pump surgery, suggesting a compensatory immunosuppressive response to inflammation. Distinct EV profiles were linked to use of CPB and may contribute to complications such as postoperative cognitive dysfunction	[7]
On-pump CABG or minimally invasive heart valve surgery: 15 patients	Plasma EVs: CD63^+^, HSP70^+^	Early rise of anti-apoptotic plasma EVs post-CPB in older adults is consistent across on-pump surgeries; exosomal content varies by surgery type. The monitoring of plasma EVs may identify ischemia-tolerant patients	[14]
Off-pump CABG: 44 patients	er-EVs: CD235^+^ l-EVs: CD45^+^ p-EVs CD61^+^	Total EV counts varied with graft type, suggesting EVs may reflect graft-specific inflammatory responses Postoperative l-EVs were significantly lower in patients with LIMA-LAD grafts than in those with RIMA-LAD grafts	(35)
TAVR: 56 severe AS patients	e-EVs: CD144^+^, CD62E^+^, CD31^+^/CD41^−^ p-EVs: CD41^+^	All e-EVs subpopulations decreased 3 months after TAVR, along with an increase in endothelial function	(36)
TAVR: 92 severe AS patients	e-EVs: CD31^+/^AnnV^+^, CD31^+^/AnnV^−^, CD31 + /CD42b^−^, CD62E^+^ p-EVs: CD31^+^/CD42b^+^	CD62E^+^ e-EV population decreased gradually, while circulating p-EVs increased gradually Determination at 1 week, 1, 3 and 6 months after TAVR	(37)
TAVR: 9 severe AS patients	e-EVs: CD31^+^/AnnV^+^ p-EVs: CD41^+^/AnnV^+^ l-EVs: CD45^+^/AnnV^+^	Levels of e-EVs, p-EVs or l-EVs do not change on the fifth day after TAVR	(38)
TAVR: 139 patients	l-EVs: CD45^+^ EVs: AnnV^+^	TAVR decreases CD45^+^ EVs, indicating reduced leukocyte activation and supporting its anti-inflammatory effect High pre-TAVR levels of AnnV^+^ EVs are associated with a > fivefold increased risk of MACCE during a median follow-up of > 10 months, independent of other clinical factors	(39)
TAVR: 53 patients TAVR-PCI: 15 patients	EVs: AnnV^+^ EVs: TF^+^ p-EVs: CD41^+^ e-EVs: CD31^+^/CD144^+^	Sustained elevation of AnnV^+^ EVs, p-EVs, and TF^+^ EVs from 7 days to 6 months post-TAVR or TAVR-PCI, associated with higher D-dimer and procoagulant activity e-EVs decreased after TAVR but increased after TAVR-PCI, likely due to PCI-induced endothelial damage Findings support reconsideration of post-TAVR antithrombotic therapy, possibly including targeting AnnV^+^ EVs, especially in TAVR-PCI patients	(40)
SAVR: 135 patients with aortic valve stenosis	Circulating EV quantification by NTA	EVs decreased significantly 24 h after surgery and recovered to preoperative levels on postoperative day 7 and at 3-month follow-up. Correlation between circulating EVs and erythrocytes, LDH and creatinine levels in peripheral blood. No significant correlation between the levels of EVs and echocardiographic parameters. The moderate prosthesis-patient mismatch and impaired left ventricular mass regression correlate with low levels of circulating EVs at 3-month	(41)
MVR: 25 patients (vs control n = 29)	e-EV: CD146^+^	Six-month post-surgery, the oxidative stress levels decreased and osteoprotegerin plasma levels significantly increased. Compared with controls, e-EVs levels were significantly higher in patients before and after the surgery	(42)

AnnV, annexin V; Bc-EVs, B cell-derived extracellular vesicles; e-EVs, endothelial EVs; er-EVs, erythrocyte EV; g-EVs, granulocyte EVs; m-EVs, monocyte EVs; p-EVs, platelet EVs; l-EVs, leucocyte EVs; CABG, coronary artery bypass grafting; CPB, cardiopulmonary bypass; LIMA-LAD, left internal mammary artery to the left anterior descending artery; MACCE, major adverse cardiac and cerebrovascular events; MVR, mitral valve repair; PCI, percutaneous coronary intervention; RIMA-LAD, right internal mammary artery to left anterior descending artery; SAVR, surgical aortic valve replacement; TAVR, transcatheter aortic valve replacement

Exosomal miR-223 plays a regulatory role by modulating IL-6 and NLRP3 levels, both of which are central mediators of CPB-induced inflammation. During CPB, platelets and erythrocytes release large quantities of miR-223-containing EXOs that downregulate pro-inflammatory signaling in circulating monocytes [43]. EVs also carry extracellular mitochondrial DNA (mtDNA), a potent pro-inflammatory molecule. A study by Baysa et al*.* [9] showed that during CPB, mtDNA is predominantly encapsulated within EVs, while nuclear DNA (nDNA) is more abundant in the plasma supernatant. The altered balance of extracellular mtDNA and nDNA correlates with immune activation and may exacerbate SIRS. These findings suggest that EVs could act as vectors for damage-associated molecular patterns (DAMPs), amplifying inflammatory cascades and worsening clinical outcomes.

During surgery, the temporary cessation and sudden restoration of coronary perfusion cause myocardial IRI, which significantly contributes to postoperative myocardial dysfunction. De facto, IRI generates reactive oxygen species, triggers apoptosis, and recruits immune cells such as macrophages and neutrophils [44]. These activated cells release extracellular DNA, which originates both from necrotic and apoptotic cells as well as from active processes like neutrophil extracellular traps. This widespread cellular activation and molecular release initiate and amplify inflammatory signaling cascades, further contributing to myocardial injury [45]. In this context, EXOs play a crucial role in modulating the response to IRI. For instance, EXOs enriched in miR-21-5p and miR-199a-3p have demonstrated protective effects by enhancing cardiomyocyte survival and reducing oxidative damage [43]. These exosomal miRNAs also show promise as early non-invasive biomarkers of myocardial injury, often preceding the rise of traditional markers like troponin I [34]. In parallel, Ge X et al. [33] showed that EVs released during IRI may transfer miR-155-5p to macrophages, driving their polarization toward the pro-inflammatory M1 phenotype via activation of the JAK2/STAT1 pathway. This process enhances the expression of neutrophil chemoattractant CXCL2, leading to increased neutrophil infiltration and amplifying inflammatory damage. Additionally, these EVs influence multiple inflammatory pathways, downregulating anti-inflammatory markers like PPARγ while upregulating STAT1, STAT3, and SOCS proteins. These molecular effects correspond with quantifiable alterations in EV quantity and composition, for instance, Urbanowicz et al. [35] observed a significant decrease in total plasma EV numbers on the first postoperative day, followed by partial recovery by the third day, with reductions more pronounced in patients undergoing arterial revascularization compared to combined arterio-venous grafts. Notably, leukocyte-derived EVs (CD45^+^) decreased particularly with left anterior descending (LAD) artery revascularization by left internal mammary artery (LIMA) grafts, correlating inversely with blood flow parameters. Erythrocyte-derived EVs (CD235^+^) also decreased, suggesting reduced thrombogenic activity while platelet-derived EVs (CD61^+^) remained stable, likely due to avoidance of CPB and hemodilution. Together, these findings indicate that both the molecular cargo of EVs and their perioperative abundance dynamically modulate inflammatory and ischemia–reperfusion responses, linking mechanistic insights with clinical observations of postoperative immune regulation.

Interestingly, EVs may also exhibit cardioprotective features in specific patient subsets. A study by Carrozzo et al*.*[14] reported that in elderly patients undergoing on-pump cardiac surgery, plasma EXOs showed an early postoperative rise in anti-apoptotic proteins, including HSP70. This was particularly evident in patients undergoing CABG or minimally invasive valve surgery. Proteomic analysis revealed that these EXOs carried a panel of 22 pro-survival proteins, including catalase, which conferred cardioprotection in hypoxia–reoxygenation models. These findings suggest that EV content is modulated by surgical type and patient comorbidity, and could serve as a functional biomarker of ischemic tolerance, especially in aging hearts. Additionally, CPB technique affects the quantity and profile of circulating EVs. Aquino et al*.* [7] showed that EV levels derived from platelets (CD41^+^), endothelial cells (CD31^+^), and B cells (CD19^+^) increased significantly more in on-pump compared to off-pump CABG. In particular, the specific EV subtype CD41^+^CD62P^+^ (activated platelet-derived) and CD39^+^CD73^+^ (Treg-derived) EVs revealed a complex immunomodulatory response, possibly reflecting a compensatory attempt to limit inflammation.

Beyond systemic inflammation and myocardial injury, EVs are also implicated in postoperative cognitive dysfunction (POCD), a frequent complication of CABG. Diagnosing POCD currently relies on recognizing symptoms and detecting blood biomarkers including S100B and neuron-specific enolase. However, the underlying mechanisms and the specific genes linked to POCD remain to be elucidated [46]. Notably, the circular RNA circRNA-089763 has been found at abnormal levels in EXOs of POCD patients. Acting as a miRNA sponge for miR-7111-3p, miR-6769b-3p, and miR-670-3p, circRNA-089763 can indirectly regulate cognitive function by affecting the expression of the IGFBP5, STC, and YWHAG genes related to cognitive functions [13].

Despite technical surgical success, graft occlusion remains a major determinant of adverse cardiovascular outcomes. Several studies showed that preoperative levels of circulating MVs predict graft patency in patients undergoing CABG surgery [47, 48]. Indeed, elevated levels of specific MVs have been associated with increased thrombin generation and graft occlusion, highlighting their potential as biomarkers for surgical outcomes. A comprehensive analysis by Camera et al*.* [11] has provided significant insights into the role of MVs in graft patency for patients undergoing CABG surgery. The study showed that high levels of specific MVs are predictive of graft failure. The study developed a scoring system based on six classes of MVs. Patients with higher scores had a greater risk of graft occlusion. This score was more effective in predicting graft patency than other individual MV classes or traditional biomarkers like D-dimer levels. Interestingly, these MVs exhibit pro-thrombotic and pro-inflammatory properties, which contribute to thrombin generation and graft occlusion. Thus, analyzing MV levels pre- and postoperatively, clinicians may potentially predict and mitigate the risks of graft failure. Overall, the study underscores the importance of monitoring MVs as biomarkers for optimizing graft outcomes and improving patient prognosis. Their detailed findings highlight the intricate interplay between MVs, endothelial function, and graft patency, providing a pathway for targeted therapeutic strategies to enhance longevity and success CABG.

### Valve repair or replacement surgery

VHD comprises a spectrum of progressive disorders that frequently require surgical correction. Although surgical and transcatheter techniques have improved substantially, patients undergoing valve interventions remain at risk for complications such as systemic inflammation, endothelial dysfunction, thrombosis, and adverse ventricular remodeling. In this context, EVs are gaining attention as both biomarkers and mediators of these perioperative events. In patients with VHD, the circulating levels of MVs derived from endothelial and platelets are consistently elevated, influencing vascular tone, coagulation, and immune activation (Table 2) [49, 50]. Interestingly, endothelial MVs disrupt nitric oxide (NO) signaling by reducing endothelial nitric oxide synthase (eNOS) phosphorylation and promoting eNOS uncoupling, thereby increasing oxidative stress and impairing vasodilation [51]. This endothelial dysfunction contributes to a pro-inflammatory and pro-thrombotic state that may worsen postoperative outcomes. Alterations in the protein composition of circulating MVs have also been linked to coagulation abnormalities and systemic inflammation in patients undergoing valve surgery with CPB [52]. A proteomic analysis identified distinct patterns of MV proteins before and after surgery, with a postoperative increase in pro-inflammatory proteins. Additionally, proteins involved in coagulation showed significant alterations following surgery, which may contribute to the activation of systemic inflammatory responses and coagulation disorders. However, a range exosomal proteins, particularly catalase, which possess cardioprotective effects were found to be significantly elevated following mitral and aortic valve surgeries [14]. Therefore, MV proteomic profiles may offer prognostic insights and reflect patient-specific perioperative vulnerability.

In the setting of mitral valve repair, EV signatures have been associated with postoperative recovery. Pizzino et al. [53] observed that elevated exosomal miR-21-5p levels during late postoperative follow-up correlated with favorable left ventricular reverse remodeling, suggesting that miRNA-enriched EVs could serve as predictive biomarkers of structural recovery. For high-risk or inoperable patients, transcatheter edge-to-edge repair (TEER) provides a less invasive alternative for treating significant mitral regurgitation. Interestingly, traditional echocardiographic parameters often fail to predict clinical outcomes post TEER. In contrast, pre-procedural exosomal miRNA profiles, including miR-133a, miR-199a-3p, and miR-221, have been shown to correlate with post-procedural improvements in function and ventricular dimensions. MiR-199a-3p, miR-590-3p, miR-150, miR-155, and miR-27 correlated with alterations in left ventricular end-diastolic diameter, while miR-590-5p and miR-25 were linked to changes in pulmonary artery pressure [54]. These exosomal miRNA profiles underscore the potential of EVs as novel biomarkers for evaluating the progression of mitral valve disease (MVD) and the response to mitral valve repair (MVR) offering a promising tool for individualized therapeutic planning.

Furthermore, in patients with mitral valve prolapse, circulating endothelial EVs were markedly elevated at baseline, reflecting significant endothelial activation and dysfunction. These elevations were accompanied by other markers of endothelial impairment and oxidative stress, including reduced α- and γ-tocopherol, elevated ornithine, diminished global arginine bioavailability ratio, and increased asymmetric and symmetric dimethylarginines. Six months after MVR, systemic oxidative stress largely normalized, as indicated by recovery of α-tocopherol levels, but endothelial EVs and osteoprotegerin (OPG) remained elevated, demonstrating that endothelial dysfunction persists despite restoration of redox balance. The sustained elevation of endothelial EVs suggests ongoing endothelial activation and may reflect disruption of nitric oxide signaling, in part due to arginase-mediated depletion of arginine. Elevated OPG levels, together with persistent endothelial EVs, may indicate endothelial-to-mesenchymal transition, a process implicated in mitral valve remodeling. Clinically, these findings highlight that circulating endothelial EVs serve as sensitive markers of residual endothelial dysfunction after MVR, and their persistence identifies patients at continued risk of vascular complications [42].

Therapeutic options for aortic valve disease (AVD) include medical therapy for symptom management and surgical or transcatheter aortic valve replacement (SAVR or TAVR) for definitive treatment in severe cases. The choice between surgical and transcatheter approaches depends on patient-specific risk factors and anatomical considerations [55]. Endothelial dysfunction, lipoprotein accumulation, chronic inflammation, and calcium nodule deposits characterize pathological aortic valves. In patients with aortic stenosis (AS), the enrichment in endothelial and platelet MVs, are important indicators of vascular damage and endothelial dysfunction. Endothelial MVs released from activated or apoptotic endothelial cells act as sensitive markers of endothelial injury, correlating closely with vascular impairment [56]. Conversely, MV derived from activated platelets play key roles in coagulation and inflammatory processes, thereby contributing to the pro-thrombotic state commonly observed in AS patients [57]. The interplay between these MV subtypes not only reflects ongoing vascular injury but also holds potential as prognostic biomarkers for identifying patients at elevated risk for adverse cardiovascular events [58]. Notably, the shear stress derived from narrowed aortic valve contributes to systemic inflammation by activating circulating blood cells, leading to the formation of MVs. In patients with severe AS, MVs derived from platelets (CD31^+^/CD61^+^ or CD62P^+^) were elevated and correlated with valvular shear stress, while elevated level of MVs from leukocytes (CD11b^+^) and endothelial cells (CD62E^+^) correlated with monocyte activation and systemic inflammation [59]. Additionally, the study conducted by Hmadeh et al*.* [60] identifies the calcified aortic valve as a significant reservoir of platelet, leukocyte, and endothelial cell EVs with procoagulant activity. Confined within the valve tissue, aortic stenotic valve-derived EVs convert valvular endothelial cells into a pro-thrombotic, pro-inflammatory phenotype, promoting immune cell recruitment, thrombogenesis, and endothelial injury. Notably, these EVs also enhance the expression of key pro-inflammatory molecules such as VCAM-1, ICAM-1, and NF-kB, while downregulate eNOS, the crucial enzyme for maintaining vascular healthy. Aortic stenotic valve-derived EVs activate a redox-sensitive signaling axis involving the AT1R/NADPH oxidase/SGLT2 pathway, leading to oxidative stress, activation of MAPKs (p38, ERK1/2, JNK), and nuclear translocation of phosphorylated NF-kB, thereby perpetuating a cycle of inflammation and endothelial dysfunction. Aortic stenotic valve-derived EVs were shown to induce expression of bone morphogenetic protein 2 (BMP-2) and SGLT2 which are factors linked with osteoblastic differentiation of valvular interstitial cells and calcification. Further, highlighting the pro-thrombotic nature of the stenotic valve, this study confirmed the presence of tissue factor (TF) and plasminogen activator inhibitor-1 (PAI-1), reinforcing earlier findings that suggest the aortic stenotic valve is a contributor to systemic thromboembolic risk, including stroke [60].

Marchini et al. (38) reported that in patients with severe AS undergoing TAVR, circulating EV levels, including endothelial- (Annexin V^+^/CD31^+^), platelet- (Annexin V^+^/CD41^+^), and macrophage-derived (Annexin V^+^/CD45^+^) vesicles, remained unchanged five days post-procedure, suggesting that EV concentrations remain stable in the early post-TAVR phase and that longer follow-up may be required to observe significant reductions [38]. In contrast, patients undergoing SAVR exhibited a transient increase in circulating EVs up to seven days postoperatively, followed by a return to baseline by three months, indicating a possible role for EVs in mediating adaptive cell–cell communication during recovery. Lower EVs levels after SAVR were associated with impaired left ventricular mass regression and the development of prosthesis-patient mismatch, suggesting prognostic potential. Overall, these findings support a role for EVs as dynamic mediators of intercellular signaling and potential biomarkers for recovery and adverse outcomes following SAVR [41].

The introduction of TAVR has transformed AVD management in high-risk patients [61]. TAVR is associated with a significant decline in circulating endothelial-derived EVs, reflecting improved vascular homeostasis. However, platelet-derived EVs may remain elevated post-TAVR, potentially due to prosthesis interaction or ongoing subclinical thrombogenesis. Jung et al. [37] studied ninety-two elderly patients who underwent TAVR through transfemoral or transapical approaches with a follow-up for up to 12 months, evaluating clinical outcomes, echocardiographic parameters, and blood samples at each interval. MV levels were significantly elevated before TAVR with a marked decline up to six months post-procedure, suggesting an improvement in endothelial function after TAVR. In contrast, platelet MV level slightly increased after TAVR and remained elevated throughout the follow-up period. This increase was attributed to interactions between platelets and the newly implanted valve or early thrombus formation on the valve cusps. The reduction in endothelial MV levels suggests that TAVR not only alleviates the mechanical obstruction caused by the stenotic valve but also restores endothelial integrity and function [37]. The study by Horn et al. [36] also demonstrated that TAVR significantly improved endothelial function, as evidenced by increased flow-mediated dilation and decreased levels of endothelial MVs. The authors found that improved stroke volume and pulsatile flow patterns after TAVR contributed to these beneficial effects. Also, the study showed that elevated wall shear stress post-TAVR was a key factor in enhancing endothelial function and reducing endothelial MV release. Additional studies further support that TAVR not only improves valvular hemodynamics but also exerts systemic vascular benefits, underscoring the value of endothelial EVs as potential biomarkers for postoperative recovery and prognosis. A significant decline in leukocyte-derived EVs after TAVR was reported, consistent with reduced leukocyte activation and an overall anti-inflammatory effect of the procedure. Importantly, high pre-procedural levels of phosphatidylserine-exposing EVs (PS^+^-EVs) independently predicted adverse cardiovascular outcomes, conferring more than a fivefold increased risk of major adverse cardiac and cerebrovascular events during follow-up [39]. These findings indicate that PS^+^-EVs reflect a procoagulant state, as externalized phosphatidylserine facilitates thrombin generation and clot formation. Consistent results were reported by Chi H et al*.* [40], who observed increases in PS^+^-EVs, platelet EVs, and tissue factor-positive EVs (TF^+^-EVs), all associated with heightened coagulation activity. Endothelial-derived EVs tended to decrease after isolated TAVR but increased when percutaneous coronary intervention was performed concomitantly, suggesting additive endothelial stress in combined procedures. Functional assays confirmed that PS^+^-EVs contribute to enhanced procoagulant activity, correlating with biomarkers of platelet and endothelial activation, thrombin generation, and fibrin formation. These findings highlight a persistent hypercoagulable state after TAVR and emphasize the role of PS^+^-EVs as both mechanistic mediators and prognostic markers of post-procedural thrombotic risk. Collectively, this evidence supports the need for refined antithrombotic strategies beyond standard dual antiplatelet therapy to mitigate EV-driven thrombogenicity and improve long-term outcomes following TAVR.

Building on these observations, Hosen and colleagues [62] further explored the prognostic role of EV molecular cargo by analyzing circulating exosomes and exosomal miRNAs in patients undergoing TAVR for severe AS. The study identified several miRNAs, including miR-122-5p, miR-26a, miR-192, miR-483-5p, miR-720, miR-885-5p, and miR-1274, that were significantly deregulated in patients without left ventricular ejection fraction (LVEF) improvement after TAVR. In particular, the high level of miR-122-5p correlated negatively with LVEF post-TAVR. By several in vitro experiments, they have demonstrated that miR-122-5p may be shuttled through large EVs from endothelial cells into cardiomyocytes, where reduces the expression of the anti-apoptotic gene BCL2, thereby decreasing the viability of recipient cardiomyocytes.

In summary, EVs are emerging as dynamic indicators of disease severity and therapeutic response in valve surgery. Their role extends beyond passive biomarker function to active modulation of inflammation, endothelial integrity, and myocardial recovery. Incorporating EV profiling into clinical workflows could enable risk stratification, guide treatment choices, and enhance postoperative follow-up and ultimately contributing to more personalized and effective care in VHD.

## Extracellular vesicles as therapeutic agents for cardioprotection

The myocardium is highly vulnerable to damage during ischemia due to its high oxygen demands and dependence on oxidative metabolism. Although reperfusion is crucial for restoring blood flow, it paradoxically causes IRI through mechanisms like pH shifts, accumulation of reactive oxygen species, calcium overload, and increased inflammation. To mitigate both ischemic and reperfusion injuries, cardioprotective strategies are critical, particularly in cardiac surgeries involving CPB and cardioplegic arrest where hypothermia and cardioplegia solutions are used to protect the heart, but challenges still remain in mitigating myocardial ischemia, reperfusion injury, and the complications of cardioplegia [63].

Beyond conventional cardioprotective strategies, several investigations have highlighted the therapeutic potential of EVs, particularly EXOs, in myocardial preservation. EXOs derived from human cardiosphere-derived cells (CDCs) and cardiac progenitor cells (CPCs) have cardioprotective properties through mechanisms including the inhibition of apoptosis, stimulation of cardiomyocyte proliferation, and promotion of angiogenesis. Animal studies have shown that administration of CDC-derived EXOs into the infarct border zone leads to reduced scar formation, increased viable myocardial mass and wall thickness, and improved overall cardiac function. Barile et al. [64] reported that treatment with human CPC-derived EXOs attenuated cardiomyocyte apoptosis and stimulated angiogenesis, contributing to enhanced post-MI cardiac function. Similarly, Chen et al*.* [15] found that delivery of mouse CPC-derived EXOs in a murine IRI model inhibited cardiomyocyte apoptosis. These effects appear to be comparable to those observed after administration of the parent CPCs themselves, indicating that CPC-derived EXOs can serve as critical paracrine effectors [65]. In addition, CDC-derived EXOs, whether secreted under normoxic or hypoxic conditions, have exhibited anti-apoptotic effects in human embryonic stem cell-derived cardiomyocytes, underscoring their therapeutic promise in the treatment of ischemic heart disease [66].

The precise mechanisms by which EXOs confer cardioprotection remain to be understood. Current evidence suggests involvement of their miRNA cargo. However, several mechanistic aspects, such as how exosomal miRNAs integrate into endogenous RNA-induced silencing complexes and function among abundant host miRNAs, require further studies. Notably, CPC-derived EXOs are highly enriched in miR-146a-3p, miR-132, and miR-210 that possess anti-apoptotic and proangiogenic activity, as shown via gain- and loss-of-function studies [64, 67]. In vitro, miR-146a mimics inhibited oxidative stress-induced cardiomyocyte death [67]. Moreover, miR-146a-knockout mice displayed larger infarct areas than their wild-type counterparts; administration of miR-146a mimics at the time of MI significantly mitigated this effect. Additionally, EXOs derived from CDCs transfected with a miR-146a hairpin inhibitor were less protective than control [64, 68]. In a rat model, CPC-derived EXO administered 48 h post-reperfusion significantly reduced infarct size, an effect attributed to the exosomal transfer of miR-181b to macrophages, leading to suppression of protein kinase C delta (PKCδ) expression. Notably, fibroblast-derived EXOs were not inherently protective, but when engineered to carry miR-181b, they displayed similar cardioprotective benefits by reprogramming macrophage function [16]. In addition, the Y RNA fragment termed EV-YF1 has been identified as abundant in CDC-derived EXOs, and its levels correlate with the cardioprotective potency. Indeed, EV-YF1 modulates macrophage phenotype within the ischemic myocardium, specifically inducing the expression of the anti-inflammatory and cardioprotective cytokine IL-10 [69].

CDC-derived EXOs have been shown to modulate the secretory phenotype of dermal fibroblasts, effectively converting them into therapeutically active cells. Specifically, fibroblasts primed with these EXOs exhibited significantly elevated secretion of stromal cell-derived factor-1 (SDF-1α) and vascular endothelial growth factor (VEGF), alongside marked alterations in the miRNA content of their secreted EVs [70]. When these primed fibroblasts were injected intramyocardially into rats one month after MI, they enhanced cardiac pump function, increased vascular density, and reduced scar mass. In contrast, unprimed fibroblasts did not have reparative effects. These findings suggest that CDC-derived EXOs can reprogram otherwise inert fibroblasts into cells with regenerative capacity, thus contributing to the long-term therapeutic benefits observed after CDC administration, probably through the activation and modification of resident fibroblasts within the injured myocardium.

In addition, ischemia-induced release of EVs into the circulation can represent a key mechanism underlying the cardioprotection observed in remote ischemic conditioning (RIC) [71]. Giricz et al*.* [72] provided the first direct evidence that EXOs can transmit the cardioprotective signals of RIC. In particular, administration of EXOs isolated from preconditioned donor hearts significantly reduced infarct size in non-preconditioned recipient hearts undergoing IRI. Notably, RIC-induced EXOs are enriched in miR-24, which is taken up by cardiomyocytes and reduces oxidative stress-induced apoptosis by downregulating the pro-apoptotic protein Bim [73]. In vivo*,* administration of miR-24-enriched EXOs attenuated infarct size and improved cardiac function in a rat model of myocardial infarction. The cardioprotective effects were abolished by miR-24 inhibitors, confirming a functional role for miR-24 in EXO-mediated myocardial protection. Complementary human studies in CABG patients further support the role of exosomal miRNAs in RIC, with increased levels of 26 miRNAs, including the cardioprotective miR-21, after RIC and coupled to corresponding reductions in postoperative troponin I levels [74]. Additionally, ischemic preconditioning has been associated with rapid changes in the mRNA content of cardiac EVs, although no significant alterations in their DNA content was observed [75].

The protein cargo of plasma-derived EXOs also plays a critical role in mediating the pro-survival effects of circulating EXOs in both ex vivo*, *in vivo, and in vitro models of IRI. For instance, the exosomal HSP70 activates ERK1/2 in cardiomyocytes, triggering toll-like receptor (TLR4) and heat shock protein beta-1 (HSPB1) phosphorylation, leading to a cardioprotective. If any of these proteins are selectively blocked or if HSP70 is absent from the EXO surface, the cardioprotective signal is not transmitted [76].

Emerging experimental evidence supports the therapeutic potential of mesenchymal stem cell-derived EVs (MSC-EVs) in promoting myocardial repair after injury. These effects may be attributed to the diverse RNA cargo, which is shaped by various endogenous and exogenous stimuli. Several miRNAs carried by MSC-EVs have been identified as key effectors in cardioprotection. Notably, miR-144-3p delivered via MSC-EVs has been shown to inhibit cardiomyocyte apoptosis and autophagy under ischemia/hypoxia conditions by targeting ROCK1 and activating the PI3K/AKT/mTOR signaling pathway [77]. Similarly, several other miRNAs such as miR-455-3p, miR-143-3p, miR-24, and miR-210 have been implicated in the regulation of cell survival pathways, suppression of oxidative stress, and inhibition of maladaptive remodeling following cardiac injury [78–81].

While most studies focus on the protective role of upregulated miRNAs, certain miRNAs appear deleterious and are downregulated by MSC-EVs to confer benefit. For instance, the long non-coding RNA (lncRNA) A2M-AS1 in MSC-EVs acts as a sponge for miR-556-5p, reducing apoptosis and oxidative stress [82]. Similarly, MSC-EVs downregulate miR-17-5p, which is upregulated during IRI, resulting in reduced cell death [83]. Another mechanism involves MSC-EV-induced upregulation of HCP5, which sponges miR-497 and consequently activates the IGF1/PI3K/AKT signaling cascade, promoting cardiomyocyte survival under stress [84]. Preconditioning of MSCs with environmental or pharmacological stimuli has emerged as a promising strategy to enhance the therapeutic efficacy of their EVs in cardioprotection. Hypoxia preconditioning modifies the molecular cargo of EVs, enhancing their reparative potential. Hypoxia-conditioned MSC-derived EXOs exhibit increased levels of miRNAs such as miR-125b-5p and miR-210, which contribute to reduced cardiomyocyte apoptosis and improved cardiac function in myocardial infarction models [85–87]. Likewise, EXOs from hypoxia-preconditioned induced pluripotent stem cell-derived cardiomyocytes or cardiovascular progenitors contain miRNA clusters (*e.g.*, miR-106a–363) and lncRNAs (*e.g.*, MALAT1) that promote cardiomyocyte proliferation, vascularization, and myocardial survival [88, 89]. Hypoxia-induced also upregulation of miR-224-5p in MSC-EVs that inhibited TXNIP, a key oxidative stress mediator, promoting myocardial repair [90].

**Fig. 2:**
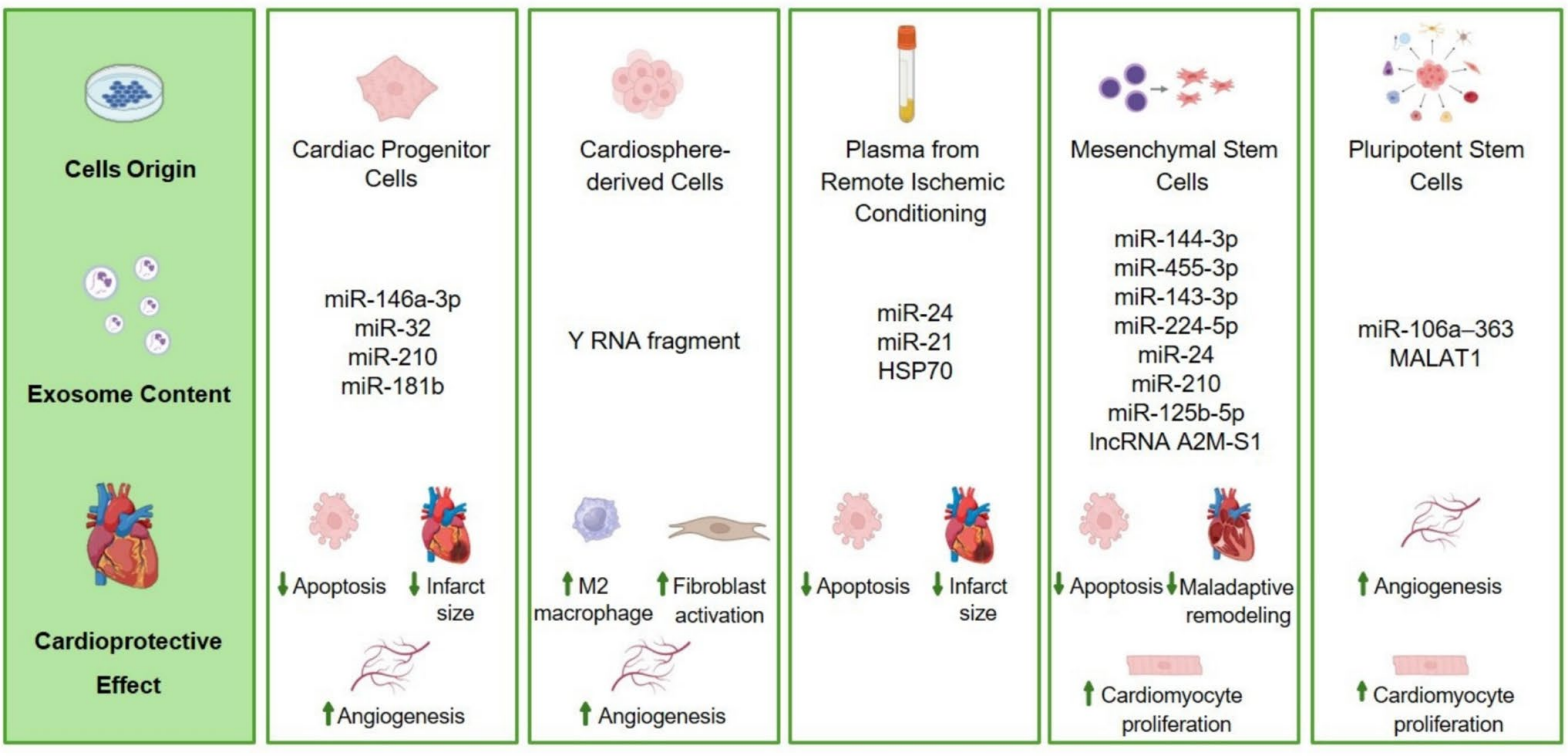
Cellular sources and cardioprotective effects of extracellular vesicles (EVs). EVs derived from cardiac progenitor, cardiosphere-derived, mesenchymal stem and pluripotent stem cells, and remote ischemic conditioning contribute to cardiac repair and remodeling modulating apoptosis, angiogenesis, or cardiomyocyte proliferation

Similar benefits have been observed with preconditioning agents, such as IFN-γ, tongxinluo, and hemin, which act through diverse mechanisms including upregulation of growth factors, suppression of pro-inflammatory cytokines, and enrichment of cardioprotective miRNAs (*e.g.,* miR-21-5p, miR-146a-5p, miR-183-5p) [91–93]. Atorvastatin preconditioning has emerged for its multiple effects. Indeed, atorvastatin-pretreated MSC-EVs promote macrophage polarization toward the anti-inflammatory M2 phenotype via miR-139-3p-mediated suppression of STAT1 [94]. Additionally, atorvastatin-enhanced EVs improve endothelial cell migration, angiogenesis, and myocardial recovery post-infarction, via lncRNA H19 regulation of miR-675 and activation of proangiogenic factor VEGF and ICAM-1. This cardioprotective effects were abrogated when lncRNA H19 was depleted in the atorvastatin-pretreated MSCs and was mimicked by overexpression of lncRNA H19 [95]. Figure 2 highlights the cellular origins, molecular contents, and mechanisms through which exosomes mediate cardioprotective effects.

## Conclusion and future directions and clinical implementation

Detailed EV signatures obtained preoperatively may offer insights into patient endothelial function, inflammatory status, and propensity for thrombotic events. Thus, the EV profile may suggest an increased thrombotic risk, which may benefit from more aggressive antiplatelet therapy or alternative surgical techniques. Additionally, monitoring changes in EV composition post-surgery can provide early indicators of complications, allowing clinicians to adjust treatment plans proactively and improve long-term outcomes. Beyond diagnostics, EV profiling is also being explored as a tool for monitoring the efficacy of therapeutic interventions. Shifts in the molecular cargo of EVs may serve as surrogate markers for the success of therapeutic regimens. This real-time feedback is particularly useful in the postoperative period, where early detection of adverse trends may prompt timely modifications in treatment.

Importantly, EVs are also being investigated as therapeutic agents in cardioprotection. Derived from various progenitor or stem cell populations, such as CPCs or MSCs, EVs carry a rich cargo of cardioprotective molecules including proteins, lncRNAs, circRNAs, and miRNAs. These components have been shown to reduce apoptosis, modulate inflammation, promote angiogenesis, and enhance myocardial repair in preclinical models of ischemia–reperfusion injury. EVs can mimic many of the beneficial effects of cell-based therapies while avoiding challenges like immune rejection or poor cell engraftment. In surgical contexts, their administration either preoperatively, intraoperatively, or postoperatively might reduce myocardial damage and support functional recovery. Their small size and ability to cross biological barriers make EVs ideal candidates for targeted delivery, and ongoing research is exploring engineered EVs with enhanced therapeutic potency or tissue-specific targeting.

The integration of EV profiling with emerging technologies such as artificial intelligence and nanotechnology holds exciting promise. The application of machine learning models to large datasets of EV signatures may uncover hidden patterns that predict patient outcomes more accurately than traditional biomarkers alone. Ultimately, decoding the molecular messages within EVs not only enriches our understanding of cardiovascular pathophysiology but also paves the way for individualized care strategies. Patients identified as having a high risk for postoperative complications based on their EV profile might receive tailored pharmacological therapies, customized follow-up protocols, or specialized rehabilitation programs designed to mitigate those risks. With continued advances in analytical technologies and the establishment of robust clinical protocols, EV profiling and therapeutic EVs are poised to become cornerstones of modern cardiovascular medicine.

## Data Availability

No datasets were generated or analysed during the current study.

## Abbreviations

AS: aortic stenosis
AVD: aortic valve disease
CABG: coronary artery bypass grafting
CAD: coronary artery disease
CDCs: cardiosphere-derived cells
CPB: cardiopulmonary bypass
CPCs: cardiac progenitor cells
EVs: extracellular vesicles
EXOs: exosomes
IRI: ischemia-reperfusion injury
LVEF: left ventricular ejection fraction
MSC-EVs: mesenchymal stem cell-derived extracellular vesicles
MSCs: mesenchymal stem cells
MVD: mitral valve disease
MVR: mitral valve repair
MVs: microvesicles
POCD: postoperative cognitive dysfunction
RIC: remote ischemic conditioning
SAVR: surgical aortic valve replacement
SIRS: systemic inflammatory response syndrome
TAVR: transcatheter aortic valve replacement
TEER: transcatheter edge-to-edge repair
VHD: valvular heart disease
